# “What if They Are Pre-conception? What Should We Do?”: Knowledge, Practices, and Preferences for Safer Conception Among Women Living With HIV and Healthcare Providers in Gaborone, Botswana

**DOI:** 10.3389/fgwh.2020.582463

**Published:** 2020-12-15

**Authors:** Sarah A. Gutin, Gary W. Harper, Neo Moshashane, Christina Bitsang, Jane Harries, Doreen Ramogola-Masire, Chelsea Morroni

**Affiliations:** ^1^Center for AIDS Prevention Studies, Division of Prevention Science, University of California, San Francisco, San Francisco, CA, United States; ^2^Women's Health Research Unit, School of Public Health and Family Medicine, Faculty of Health Sciences, University of Cape Town, Cape Town, South Africa; ^3^Department of Health Behavior and Health Education, School of Public Health, University of Michigan, Ann Arbor, MI, United States; ^4^Botswana-UPenn Partnership (BUP), Gaborone, Botswana; ^5^Botswana Harvard AIDS Institute Partnership, Gaborone, Botswana; ^6^Career and Counseling Services, University of Botswana, Gaborone, Botswana; ^7^Department of Obstetrics and Gynecology, Faculty of Medicine, University of Botswana, Gaborone, Botswana; ^8^Liverpool School of Tropical Medicine, Liverpool, United Kingdom

**Keywords:** safer conception, childbearing, reproductive rights, stigma, Botswana, women living with HIV (WLHIV)

## Abstract

Safer conception interventions that address HIV care, treatment, and prevention for HIV-affected couples are increasingly available in sub-Saharan Africa. Botswana, an HIV endemic country, is yet to offer formal safer conception services although universal test-and-treat approaches mean that increasing numbers of young, sexually active people living with HIV will start treatment and likely desire childbearing. In order to advance the safer conception discussion in Botswana, it is necessary to understand the current safer conception knowledge, practices, and preferences of healthcare providers and women living with HIV (WLHIV). We conducted qualitative in-depth interviews with 10 HIV healthcare providers and 10 WLHIV in Gaborone. Interviews were analyzed using a phenomenological approach. Safer conception knowledge was limited and safer conception discussions were rare. Healthcare provider and WLHIV preferences were at odds, with providers preferring WLHIV to initiate safer conception discussions, and WLHIV desiring providers to initiate safer conception discussions. Quotes from women and providers highlight deeper issues about power dynamics, concerns about stigma among women, and provider fears about promoting pregnancy. Providers emphasized the need for guidelines and training in order to improve the provision of safer conception counseling. These findings point to areas where safer conception in Botswana can be improved. Both WLHIV and providers would benefit from having information about a range of safer conception methods and approaches. In addition, since WLHIV felt hesitant about initiating safer conception conversations and feared stigma, and because putting the onus for starting safer conception discussions on women is a reversal of normal roles and power structures, providers must take the lead and routinely initiate fertility desire and safer conception discussions. Assisting healthcare providers with clear safer conception guidelines and training would improve the provision of accurate safer conception counseling and facilitate reproductive choice.

## Introduction

Across various sub-Saharan African countries, between 12 and 64% of women living with HIV (WLHIV) report a desire for a future pregnancy, making the risks for HIV transmission to partners and infants a concern (1–4). Although prevention of mother-to-child transmission of HIV (PMTCT) interventions are highly effective (5, 6), safer conception methods and approaches have been under-utilized. A variety of safer conception strategies exist including ART to reduce the infectiousness of the partner living with HIV (7, 8), pre-exposure prophylaxis (PrEP) for uninfected partners (9–11), condomless sex limited to the time of peak fertility (12), and artificial insemination (13, 14). Although some safer conception methods are cost-prohibitive, others are low technology and are more readily available in resource-constrained settings. Safer conception programs are still nascent in many high HIV prevalence countries but are gaining momentum (15–20).

One possible reason for poor safer conception utilization in sub-Saharan African settings is low levels of knowledge about safer conception techniques among both providers and clients (21–24). As a result, safer conception is rarely discussed during health consultations and WLHIV often do not seek safer conception counseling (24, 25). Despite the general lack of safer conception information during consultations, research has shown that WLHIV desire strategies to reduce HIV risk during conception and are receptive to provider discussions about safer conception (23, 26, 27). This suggests that safer conception advice offered by providers may be well-accepted by WLHIV as a way to make conception and pregnancies safer.

Even in supportive healthcare contexts, studies from sub-Saharan Africa suggest HIV care providers do not routinely discuss fertility desires or initiate accurate safer conception counseling with WLHIV of reproductive age (15, 16, 21, 23, 24, 27, 28). This may be a byproduct of many decades of HIV prevention messaging that has stigmatized childbearing among WLHIV, focused on condom use, and discouraged WLHIV from having repeat pregnancies (4, 24, 27, 29–32). As a result, communication is inhibited because women are often afraid to discuss their childbearing desires and anticipate stigma from healthcare providers (29, 33, 34). In addition, power imbalances exist between healthcare providers and clients, which makes it difficult for WLHIV to initiate these discussions (35, 36). Research from South Africa and Uganda suggests there is a need for routine provider initiation of counseling regarding future pregnancy (24, 27, 37).

Botswana has signed on to the UNAIDS 95-95-95 targets (95% HIV counseling and testing, 95% ART initiation, 95% viral load suppression) and has reported considerable progress toward meeting international targets. However, the HIV incidence rate in the country indicates substantial ongoing transmission (38). With an HIV prevalence among women of reproductive age of 24.6% (39) in Botswana and local data showing that 60% of women knew that they were living with HIV before becoming pregnant (3), safer conception is an especially relevant approach. Despite having high HIV treatment coverage and a healthcare system where access to primary care is not a limitation (40), current policies have provided limited guidance on offering safer conception for WLHIV and safer conception services are not yet being offered in a systematic way (41). The most current Botswana HIV guidelines mention various safer conception approaches but do not detail the package of services to offer WLHIV who want to become pregnant (41).

To advance discussion about safer conception in Botswana, it is necessary to understand the current state of safer conception counseling. We conducted qualitative interviews to better understand the knowledge, practices, and preferences of healthcare providers and WLHIV about safer conception as the opinions and preferences of both groups are critical to safer conception implementation and uptake. In addition, we include recommendations for how Botswana can improve the provision of safer conception counseling. Providing safer conception services that support the health of WLHIV, their partners, and their infants is an important approach that can prevent the spread of HIV and also support the reproductive rights of WLHIV.

## Methods

### Setting, Study Population, and Participant Recruitment

We conducted 20 individual in-depth, semi-structured interviews between August 2015—January 2016 with HIV/sexual and reproductive healthcare (SRH) providers and WLHIV in Gaborone, Botswana. Providers and WLHIV were drawn from six government-supported clinics, non-governmental organization-supported clinics, and a clinic at a tertiary educational institution in Gaborone that were all providing SRH and HIV care. The eligibility criteria for providers included being a medical doctor, nurse, or midwife, age 18 years or older, willing to participate in the study, able to give informed consent, and working at a study clinic. Eligibility criteria for women included being 20 to 40 years old, self-report of living with HIV, previously or currently pregnant, accessing care at a study clinic, willing to participate in the study, and able to give informed consent.

Providers were purposively sampled to represent a range of different clinic settings where WLHIV might access safer conception services, such as government-supported clinics and non-governmental organization-supported clinics. Providers were approached in person at their health facilities and assessed for interest and eligibility. All providers who were approached agreed to take part in the study. WLHIV were sampled to represent those who might access safer conception services in the public sector [within their reproductive years (age 20–40 years), varying amounts of time on ART, varying relationship statuses (married, in relationship, single), and varying partner status (sero-concordant or sero-different relationships)]. WLHIV accessing care at study clinics were informed about the study by health center staff and referred to the study coordinator. The study coordinator screened women for eligibility and explained the study aims. After assessing eligibility, <5% of eligible women did not complete interviews, most commonly citing time constraints. Women were reimbursed 30 Botswana Pula (~ 3 USD) to cover local transport costs. By using this sampling technique, we were not trying to create a representative sample. Rather, we were attempting to gather in-depth information that could capture the lived experiences and viewpoints of providers and women living with HIV (42).

Ethical approvals were obtained from the University of Michigan Health Sciences and Behavioral Sciences Institutional Review Board (Ann Arbor, Michigan), the University of Botswana Research Ethics Committee, the Office of Research and Development (Gaborone, Botswana), and the Health Research and Development Division of the Botswana MOH. Permissions were also obtained from heads of health facilities before recruitment of providers and women living with HIV took place. Since the only record linking the participant and the research would be the consent document, we received a waiver of documentation of written informed consent in order to fully protect the identities of all study participants. However, all participants provided comprehensive verbal informed consent.

### Data Collection and Analysis

The data collection and analytic processes were informed by a social constructivist framework (43). Such a framework focuses on capturing and reporting multiple experiences, norms, and perspectives so as to develop an enhanced and deepened understanding of a particular context and cultural setting. A social constructivist approach allows one to learn about a culture-sharing group from the perspective of the group members using the language or phrases that they use to construct meaning (44). The goal of these interviews was to understand the current knowledge, practices, and preferences of healthcare providers and WLHIV about safer conception.

The initial interview guide was drafted, tested and revised through a collaborative process involving experts in the field of SRH and safer conception (two HIV/SRH researchers from the USA and one medical doctor from Botswana), and two local researchers in Botswana with many years of experience in HIV/SRH research, to ensure exploration of appropriate constructs. All members of the study team conduct behavioral research focused on SRH among people living with HIV.

In-depth semi-structured interviews with HIV/SRH providers were conducted in English (the language of medical professional activity in Botswana) by SG in private spaces where the providers worked or in a private location of the participant's choosing. All providers were fluent in English. Interviews lasted ~ 1 h. Local, female research team members, who were fluent in both English and Setswana (the local language) conducted semi-structured interviews with WLHIV. Local research team members were experienced qualitative interviewers with topical expertise in HIV/SRH. Interviews with women took place in Setswana, English, or a mix of both, depending on participant preferences. Interviews were conducted in a private space at the health center where the woman was recruited or a private location of her choosing. Interviews lasted ~ 1 h. A transcript in English was produced for each interview from either English or Setswana digital recordings. A study team member reviewed each transcript for quality and accuracy and corrections were made when necessary.

Data were analyzed using a psychological phenomenological framework (44, 45). Psychological phenomenology is focused on describing what a group of people has in common as they experience a particular phenomenon. It is primarily an inductive analytic approach that allows patterns, themes, and categories of analysis to emerge from the data (44, 45). It is different from other qualitative approaches in that it focuses on identifying elements of a particular phenomenon by describing what the phenomenon is and how it is experienced by a group of people (45).

After reading all transcripts and creating memos, we identified significant statements in the data and grouped these into clusters of meaning and recurring themes (46). We iteratively developed inductive codes that emerged from the data to complement our initial a priori codes, which were derived from the research questions (47). Through an iterative process, SG and an expert in the field of HIV/SRH identified, discussed, and compared key themes and then developed a codebook.

The web application Dedoose (www.dedoose.com) was used to facilitate systematic data management and coding (48). After exploring and coding the main themes in each interview, cross-case and comparative analyses were conducted to expand our understanding by examining similarities and differences across cases and between groups (WLHIV vs. healthcare providers) (45, 47). SG led the analysis and consulted regularly with co-collaborators to discuss interpretation of the data and ensure the cultural salience of findings. In cases where there was disagreement about interpretation, discrepancies were discussed until consensus was achieved.

## Results

### Demographics

Ten interviews with HIV/SRH healthcare providers, and 10 interviews with WLHIV were conducted (Table 1). HIV/SRH providers consisted predominantly of nurses/midwives (nine women and one man). The mean age of providers was 41 years (ranging from 30–55 years). Providers had served people living with HIV for a mean of 10 years (ranging from 7–15 years). The mean age of WLHIV was 32 years (ranging from 24–39 years) and women had known their HIV status for a mean of 7 years (ranging from 1–13 years, although one woman had been perinatally infected). Two women were single, three were in relationships with a regular partner (not cohabiting), three were cohabiting with a regular partner, and two were married. Seven women reported their most recent sexual partner was living with HIV while three reported being in discordant relationships. Four women were pregnant at the time of their interview (none of these was a first pregnancy) while six were recently pregnant. Nine of the 10 women had become pregnant since learning their HIV status.

**Table 1:** Demographic characteristics of recruited participants.

**Women living with HIV (*n* = 10)**	
Mean age (range)	31.9 (24–39)
Relationship status	
Single	2
In relationship	3
In relationship/cohabiting	3
Married	2
Mean years since HIV diagnosis (range)	7 (1–13)
Currently on ART	10
Mean years using ART (range)	5.9 (1–13)
HIV status of primary partner	
HIV-positive (sero-concordant)	7
HIV-negative (sero-discordant)	3
Mean number of pregnancies (range)	2.4 (1–4)
Mean number of living children (range)	1.8 (1–4)
Has had pregnancy after learning HIV-positive status	
Yes	9
No	1
Currently pregnant	
Yes	4
No	6
**HIV/sexual and reproductive health providers (*****n*** **= 10)**	
Mean age (range)	41.1 (30–55)
Clinical cadre	
Nurse	6
Midwife	3
Doctor	1
Mean years as a clinician (range)	17.7 (7–30)
Mean years at clinic (range)	5.5 (1–19)
Mean years working with people living with HIV (range)	9.9 (7–15)

### Overview

In this section, we first describe (1) knowledge about safer conception methods among both providers and WLHIV. Next, we examine (2) current practices related to fertility and safer conception discussions during counseling or clinical care visits. That is followed by a description of (3) preferences for offering safer conception counseling. We end by describing (4) recommendations from healthcare providers on what they believe they need in order to implement effective safer conception services for WLHIV in Botswana. Representative quotes are provided throughout to provide richer detail and examples of the various categories and themes. When describing participants, age is listed as a range in order to protect anonymity.

### Knowledge About Safer Conception

**Knowledge about specific safer conception methods**. Both providers and WLHIV had some knowledge of the concept of safer conception and specific safer conception methods. All providers were aware of at least one safer conception method. Almost all providers discussed HIV viral suppression, half discussed insemination techniques, and half mentioned timed unprotected intercourse during a woman's fertile days. When providers had safer conception information, they explained that they had learned such information from other providers or the internet but had not received any formal training. Amongst WLHIV, the most commonly mentioned safer conception approach was also viral suppression but few were aware of other safer conception approaches. Three women mentioned timed unprotected intercourse during a woman's fertile days and one woman mentioned insemination. Some WLHIV and providers described the importance of the combination of viral suppression and timed unprotected intercourse as a valuable safer conception strategy.

### Current Practices Regarding Safer Conception and Fertility Conversations

In discussing current practices related to safer conception in Botswana, respondents focused on four main areas: (1) the frequency of safer conception discussions, (2) the timing of safer conception discussions, (3) the focus of fertility-related conversations, and (4) the reality that few women arrive for safer conception counseling.

**Frequency of safer conception discussions**. Safer conception discussions were reported to be rare by both WLHIV and providers. Over half of the women reported that neither a provider nor they themselves had ever initiated a safer conception discussion. Three providers reported that they routinely ask clients about their fertility desires. Only four providers said they had ever initiated safer conception discussions with WLHIV. However, most noted that when it was discussed, clients most often raised the topic of safer conception.

I include that (safer conception information) especially pre-test (during HIV pre-test counseling), especially if it's a young patient. I usually bring the issue of having children because I know that would be at the back of their minds so I would bring it pre-testing … But not that it comes very easily. Sometimes I remember it, but most times I just forget to talk about it but I try to talk about it post (during post-test counseling). But mostly it's from patients (the patients initiate the conversation). (Nurse midwife, 45–49 years).

We went to the nurse's office and sat down with her. I told her that we would like to start a family and that we are both living with HIV. (WLHIV, 25–29 years).

**Timing of safer conception discussions**. Healthcare providers reported that when safer conception information was proactively provided, it was often brought up at ART initiation or during adherence counseling. If it happened, this was usually a once off conversation. Women reported that it was common during these visits for providers to tell clients to come seeking care if they desired a pregnancy.

It is something that normally when I do an adherence counseling before they start treatment, I would also say it to them to say, “if you want to be pregnant this is what you should do”. (Doctor, 40–44 years).

They said if I wanted to be pregnant, I need to see a doctor before getting pregnant so that the doctor can give me a go ahead to be pregnant or tell me how long I have to stay before getting pregnant, or when to avoid getting pregnant. (WLHIV, 30–34 years).

**Focus of fertility-related conversations**. Both providers and women noted that fertility discussions tend to focus on condom use and pregnancy prevention and do not often include safer conception counseling. Some providers felt that they did not want to encourage WLHIV to continue childbearing. Perhaps because of this, few women came to seek safer conception advice.

They just advise you to use condoms and things like that, but all in all, going deep, like I said, it is not done. (WLHIV, 35–39 years).

I think we push them toward condoms … I think we just assume they should use condoms and maybe that's why they come back pregnant again even after condoms, condoms, condoms. I think so. I think even … they (WLHIV) just feel okay, we are expected to use condoms so why should I even go there and start talking of (pregnancy). (Midwife, 45–49 years).

**Few women arrive for safer conception counseling**. Providers were frustrated because they told WLHIV to come for safer conception advice and yet they acknowledged that few women came for counseling prior to pregnancy. Women also confirmed that providers told them to come for safer conception advice when they desired a pregnancy. However, most women explained they had not gone to seek safer conception advice from healthcare providers prior to becoming pregnant and instead arrived for care once they were already pregnant. Some women said they had not gone to seek safer conception advice because their pregnancies were unplanned, others because they were concerned about judgmental and negative attitudes from healthcare providers, and still others because they did not know that there were any safer conception approaches that providers could offer them.

No, it's not common, they don't ask (for safer conception advice)—usually they will just come pregnant. (Nurse, 30–34 years).

I have many children and I am afraid they will think I am irresponsible you know (if she comes seeking safer conception advice). But maybe that is not true, but I just feel they will think I don't care about myself—to have babies when taking treatment. (WLHIV, 35–39 years).

### Preferences for Offering Safer Conception Services

The preferences among providers and women for how to offer/who should initiate safer conception discussions were generally at odds. WLHIV preferred providers to initiate safer conception discussions while providers felt that it would be better if WLHIV initiated these discussions. However, each group felt that some of the onus for discussing safer conception fell on them. However, the quotes from women and providers suggest deeper issues about power dynamics, concerns about stigma among women, and provider fears about promoting pregnancy. We first discuss the perspectives of women followed by the perspectives of providers and finish by discussing the shared sentiment among providers and WLHIV that they would both feel comfortable discussing safer conception if the topic was brought up.

**Perspectives of WLHIV**. When they were asked about who should initiate safer conception conversations, women discussed (1) who should initiate safer conception discussion, (2) why they think one group or another should start these conversations, and (3) concerns about anticipated stigma and how this impacts discussion with healthcare providers. WLHIV were split on whether healthcare providers or both women and healthcare providers should initiate safer conception discussions. Some women felt that it was the responsibility of healthcare providers to bring up the topic of safer conception saying that it was difficult for women to discuss personal issues or that many women were afraid to initiate such discussions. As one woman explained, she felt it was the responsibility of the healthcare provider to ask her about fertility desires so as to “make her talk.” As a client, she felt that she could not begin conversations about intimate issues. However, some women recognized that healthcare providers do not know when women want pregnancies, and therefore felt that both WLHIV and providers could initiate safer conception discussions.

The healthcare worker should ask me if I am considering having babies. It should come from the healthcare worker. (Woman living with HIV, 35–39 years).

I think it is their (healthcare provider) responsibility (to ask about safer conception), but I think I also have to ask, because it's not like they can tell if I want to have more children or not—it may also help if one tells them. (Woman living with HIV, 25–29 years).

An important component for women, related to who should initiate safer conception discussions, was concern about negative reactions and judgmental attitudes from providers. WLHIV felt that many women were afraid or shy about asking healthcare providers about safer conception. Women felt that having providers begin these discussions would feel more comfortable because many WLHIV fear bringing up sensitive topics with their providers.

I honestly think a lot of women are scared to ask or initiate conversations with nurses, just like I was. (WLHIV, age unreported).

**Perspectives of healthcare providers**. When providers were asked about who should initiate safer conception discussions, their responses focused on two main themes: (1) a preference for WLHIV to initiate safer conception discussions because this showed investment in safer conception and (2) a recognition that they should initiate safer conception discussions because some clients may fear negative responses and judgmental attitudes from providers.

Most providers said they would feel more comfortable if WLHIV initiated safer conception conversations. Many felt it was awkward to ask women if they desired a pregnancy. Some providers thought that by initiating safer conception discussions, they would be promoting pregnancies for WLHIV and that was something they did not want to do. However, if women came seeking safer conception advice, providers felt this proved that women were dedicated to having safer conception information and were more likely to follow provider advice.

I know some people are very, very uncomfortable with that (asking about safer conception needs)—it's like they're (healthcare providers) encouraging them (WLHIV) to go and get pregnant while they're positive. (Midwife, 45–49 years).

I feel okay because if they (WLHIV) start the topic, I think they are there—they want that information (about safer conception) so just tell them, now we are going to call a spade a spade, we are going to talk about this, then you sit down, you talk about that. I don't have to hide anything. Especially if they came to me and asked for help, I think they would be willing to know everything. (Nurse, 30–34 years).

I would say it will be more comfortable if the patient initiated it (safer conception discussions) because it will appear to me that ok, the patient is comfortable with this as well but then I know as a health worker that my role is to go beyond whatever the concern for that day is, so sometimes I just need to ask. (Nurse, 30–34 years).

Some providers echoed what WLHIV said and recognized that fear of negative reactions from judgmental providers may cause some women to avoid discussions about their desire for children. Some providers felt that if healthcare providers did not start safer conception discussions, that WLHIV would not actively ask about safer conception. Despite possible discomfort or awkwardness, a number of providers recognized that they needed to put their own feelings aside and proactively ask clients about their fertility desires in order to create an environment where clients felt comfortable and free to discuss their pregnancy intentions.

Sometimes patients they will be scared to ask you. Ah, what will she (the nurse) say? So it's better to say it out so that you give them that atmosphere of feeling so comfortable to discuss these things. Where will they discuss them if they don't discuss them with you as the healthcare provider? (Midwife, 35–39 years).

If you don't initiate things they will just keep quiet. They will fear to ask. They will be shy to ask and thinking that because I am HIV-positive maybe if I talk about pregnancy, maybe they (healthcare providers) are going to say something. Maybe I won't be doing the right thing. So I think the health provider should ask because we really need to assess every part of this person. (Nurse, 55–59 years).

**Shared comfort with discussing safer conception**. Despite varying opinions about whom should start safer conception discussions, both healthcare providers and WLHIV agreed that they would feel comfortable discussing safer conception if the topic was brought up. Many WLHIV desired safer conception discussions and felt such conversations should be routine. Women felt that such information was critical to know from a young age or right after learning one's HIV-positive status.

I personally don't have any problem with that (discussing safer conception needs). I just feel okay, it's just fine.” (Midwife, 45–49 years).

I think it (safer conception discussions) should be done all the time. (WLHIV, 35–39 years).

This education should be shared with every woman. Like maybe as soon as … a woman turns 18 it should be something that she is told at every clinic visit she goes to even if she has a headache. Or maybe even as soon as a woman tests positive it should be part of the post-counseling. She should be afforded a chance to digest her results but somehow given information about carrying on as a woman which includes having children. (WLHIV, age unreported).

### Healthcare Provider Recommendations for Improving Safer Conception in Botswana

Providers offered suggestions about how safer conception services could be improved in Botswana. Key recommendations focused on (1) the need for clear guidelines and protocols around safer conception and (2) the need for formal training.

**The need for safer conception guidelines**. Providers commented that there were no safer conception guidelines and overwhelmingly discussed the need for guidelines that would outline the services they should offer to couples. Although providers felt current guidelines were very clear about what to offer pregnant WLHIV, the current guidelines were unclear when it came to pre-conception. One provider suggested that perhaps women do not seek pre-conception advice because they know that there are no clear guidelines about what to offer them. Providers felt that having clear guidelines would help them know they were offering WLHIV accurate information and the correct package of services.

There's no protocol. We need the Ministry of Health to develop that protocol or guidelines for if people … are HIV-positive or discordant and they want to conceive, these are steps you healthcare providers should take … We are waiting for that … It would be very helpful because you only have (guidelines) for those who are pregnant … we have to start them on treatment. What if they are pre-conception? What should we do? … Few, one out of twenty … will come for pre-conception counseling advice because they know there's no straight guidelines.” (Midwife, 35–39 years).

**The need for safer conception training**. In the absence of formal safer conception guidelines, providers were doing their best to share the safer conception information they had with WLHIV. However, most providers felt unprepared to discuss safer conception techniques because of the lack of clear safer conception guidelines or protocols. All providers expressed the desire for formal trainings about safer conception methods, approaches, and the correct package of services to offer WLHIV who wish to become pregnant.

If you don't have information sometimes you rely on something, maybe your research and you don't even know if it's accurate. Like sometimes you research about something on the internet but you don't even know if it's the right thing. I believe we should be trained so that when you talk to a client, you know what you are talking about. (Nurse, 35–39 years).

## Discussion

In this qualitative study, we sought to gain a deeper understanding of the knowledge, practices, and preferences of healthcare providers and WLHIV about safer conception in Botswana. We found that safer conception knowledge is limited, safer conception discussions are rare, and WLHIV would like providers to initiate routine safer conception counseling. Many women were concerned about stigma and feared bringing up sensitive topics with their providers. Providers voiced a need for clear guidelines and desired training on this topic. The results show that in order to offer effective safer conception counseling in Botswana, some fundamental changes are needed. A multi-pronged approach, that addresses limitations at the individual, interpersonal, and policy level, may be best suited to create lasting change.

### Limited Safer Conception Knowledge and Conversations

Both WLHIV and providers exhibited limited knowledge of safer conception methods. Other sub-Saharan African studies have also reported similar findings (15, 23, 27, 49, 50). By far, the safer conception method that was mentioned most often was viral suppression for the person living with HIV. This finding is similar to studies from Kenya and South Africa that have found that treatment adherence for viral suppression was understood as a safer conception approach (17, 23). This understanding of the importance of treatment adherence is encouraging and may be due to Botswana's efforts to achieve the goal of 73% virologic suppression among people living with HIV,in line with UNAIDS targets (38, 51). While this level of knowledge about the importance of viral suppression is encouraging, providers and clients would benefit from having information about a wider range of safer conception methods since one approach will not work for all couples.

Despite advice from healthcare providers that they should seek pre-conception care, few WLHIV were arriving for safer conception counseling. This created a clear tension between the recommended medical advice women were receiving and the fact that most women do not arrive for care until they are pregnant, thus forgoing safer conception. As noted here and in other settings, providers often emphasize a condom-centered prevention approach (24, 27). This likely implies to WLHIV that pregnancy is not encouraged and inhibits women from seeking pre-conception support.

### Power Dynamics Between WLHIV and Healthcare Providers

WLHIV and healthcare provider views on who should initiate safer conception discussions are generally at odds, with WLHIV wanting providers to initiate these conversations, and providers feeling more comfortable with women initiating them. However, the expectation among healthcare providers that WLHIV should initiate safer conception discussions when they want to conceive is surprising as it is a role-reversal from how medical discussions normally begin in this setting. Sub-Saharan African data suggests the client-provider relationship is highly unequal in terms of social power with services often offered in a top-down way (35, 52). In these situations, providers wield considerable control, as it is presumed that providers know more than clients and because they often act as gate-keepers to services (15, 35, 53). This is especially true of the client/nurse relationship which is reported as particularly disempowering (52). Therefore, expecting clients to challenge the standard provider/client script and to begin safer conception discussions is a reversal of normal roles and power structures.

In addition, consistent with our research and similar to other settings, WLHIV in this study anticipated stigma and were afraid to discuss fertility desires with healthcare providers because they feared poor treatment and judgmental behaviors from providers because of their desire to have children (25, 33, 36, 54). In addition, some healthcare providers were not initiating fertility-related discussions with women because they did not want to encourage childbearing among WLHIV. Although this may stem from concerns about HIV transmission to partners and infants, not wanting to discuss safer conception may be rooted in stigmatizing concerns about promoting pregnancy amongst WLHIV. An important component of stigma is that certain groups are devalued. This differential valuing also pertains to the reproduction of the stigmatized group so that their fertility is devalued compared to other women by those with social or political power (55). Historically, there is a well-established atmosphere of stigma surrounding childbearing among WLHIV from both healthcare providers and community members (15, 29, 30, 56, 57). This acts as a cue in the environment that tells WLHIV that they, and their fertility, are not valued, and this reinforces social inequalities.

These finding highlight the link between social power and stigma. Structural and individual level stigma reinforces differential power relationships between healthcare providers and WLHIV and can be linked to forms of social power that reproduce inequalities that marginalize certain groups (58–60). Therefore, expecting people who are part of a vulnerable, marginalized, and stigmatized group to initiate a conversation about a stigmatized topic, such as childbearing among WLHIV, and to challenge well-established power structures and social hierarchies with the people they depend on for essential healthcare, is implausible.

If the advice that WLHIV should come seeking safer conception counseling is not yielding results, it is time for a new approach. Given the well-established atmosphere of stigma surrounding childbearing amongst WLHIV, and women being fearful to discuss fertility desires with healthcare providers, it falls on healthcare providers to create a welcoming environment where fertility desires and safer conception can be discussed openly (22, 33). Therefore, healthcare providers need to routinely initiate conversations about fertility desires and safer conception and reassess these desires over time since fertility desires are not static. If providers do not initiate these conversations they are unlikely to occur. Some providers recognized that it was their role to begin these discussions but others require support and values clarification training related to reproduction among WLHIV in order to separate their personal feelings from their required job functions (61). In addition, tools need to be developed that will facilitate routine screening of fertility intentions so WLHIV can be supported with either appropriate safer conception or family planning methods. Existing tools such as the One Key Question® initiative, which screens women of reproductive age by asking, “Would you like to become pregnant in the next year?”, could be adapted and may be a simple way to routinely and proactively assess pregnancy intention in a non-judgmental way (62, 63). Visual aids that can be used to assist women in understanding the various safer conception strategies would also be helpful.

### Healthcare Policy and Guidelines

As a first step, policy guidelines that instruct healthcare providers to routinely discuss fertility desires and offer safer conception or FP services, as appropriate, are needed. Other countries in the region, such as South Africa, have developed guidelines that encourage providers to routinely discuss safer conception with WLHIV of reproductive age (13, 64). Such a guideline has the added benefit of destigmatizing and normalizing such discussions. However, these guidelines need not single out WLHIV and could instead instruct all primary healthcare providers to routinely discuss fertility desires with all people of reproductive age, regardless of HIV status. Such a policy or guideline can have important structural ramifications by signaling that the reproduction of all women is equally valued. Within health centers, such a policy can change healthcare provider behavior and the way that providers interact with WLHIV and all people of reproductive age more generally. At the level of individual client interactions, it is hoped that the policy change will indicate a more accepting and non-judgmental healthcare environment where WLHIV and providers can openly discuss childbearing desires. Since providers will be expected to ask WLHIV about their fertility aspirations at each visit, this should signal that this is a normal conversation to have and that healthcare providers are receptive to discussing fertility desires.

The policy should be accompanied by guidelines that provide clear information for providers about the care that should be offered to WLHIV who wish to conceive and practical information and services that can be offered to reduce the risks of HIV transmission. Providers voiced this same recommendation as a way to improve the provision of safer conception services in Botswana. They requested clear safer conception guidelines that outline the services they should offer to WLHIV who wish to conceive and they desired training on these guidelines and safer conception more broadly. This desire among healthcare providers for policy guidelines and safer conception training has been noted in other countries as well (17, 28, 61, 65). In the absence of clear guidelines, providers are relying on whatever safer conception information they have been able to gather from various sources. However, providers wanted definitive guidance from their Ministry of Health so they would have assurance that they were offering accurate information. As recommended by other researchers, implementation guidelines that are practical and measurable along with comprehensive training with a strong education and counseling component should help with safer conception service provision (37, 65).

### Strengths and Limitations

This study has strengths and limitations. Data were drawn from a modest sample of urban healthcare providers and WLHIV in Gaborone, Botswana. This likely has implications for the applicability of the findings to rural settings. However, recruitment of participants was from six clinics in Gaborone, covering a range of settings. In addition, due to the modest sample, important attitudes may have been missed but women living with HIV and providers repeated the same themes, despite the small sample. The healthcare providers in this sample consisted mostly of nurses and midwives. The attitudes of this group of healthcare providers may differ from higher or lower-level cadres but since nurses provide the bulk of primary healthcare in Botswana (66), the attitudes of this group are especially salient. This study could have benefitted from the inclusion of male partners. The knowledge, practices, and preferences of male partners around safer conception will be important to document in future studies since most decisions about safer conception are made as a couple and men often play a dominant role in childbearing decisions in many sub-Saharan African contexts. However, in this study, we had concerns about potential disclosure challenges when trying to recruit male partners. However, women access SRH services more frequently than men, making their perspectives especially important and relevant to this topic. At the time of this study, safer conception services were not routinely offered in public sector clinics. Due to this, it may be that knowledge levels were particularly low. However, these results document the current state of safer conception services in Botswana in the absence of a formal Ministry of Health and Wellness supported service. Finally, women in this study were interviewed at various times either during or following their pregnancies. It is possible that perceptions may vary depending on the amount of time since the pregnancy, creating issues with recall. However, interviewing women at various points in their pregnancies or post-partum allowed us to examine the diverse attitudes of WLHIV who recently experienced pregnancy.

## Conclusion

By detailing the knowledge, practices, and preferences of healthcare providers and WLHIV, this work creates a starting point for additional discussions about how to best implement safer conception in Botswana. The results indicate that training about safer conception techniques will be needed for healthcare providers and informational campaigns that explain various safer conception methods will be needed to reach WLHIV. In addition, given the power differentials between WLHIV and providers, and fear among WLHIV about approaching providers about childbearing, the onus falls on healthcare providers to routinely initiate conversations about fertility desires and safer conception. Although providers may feel uncomfortable initiating safer conception conversations, they must focus on providing non-judgmental SRH services because anticipated stigma may keep WLHIV from accessing the full cascade of HIV prevention, care, and reproductive health services. Offering safer conception services in Botswana would be a valuable addition to a comprehensive HIV prevention strategy and supports the reproductive rights of WLHIV. Furthermore, by discussing fertility desires with all people of reproductive age repeatedly over time, a policy shift could signal that it is normal for all people, irrespective of HIV-status, to have reproductive aspirations that deserve to be respected and validated. By reducing HIV-related stigma surrounding reproduction and providing care and services that are free from judgment, it is also possible to reinforce and embrace a human rights framework that recognizes the basic right of all couples and individuals to decide freely and responsibly the number, spacing, and timing of their children.

## Ethics Statement

Ethical approvals were obtained from the University of Michigan Health Sciences and Behavioral Sciences Institutional Review Board (Ann Arbor, Michigan), the University of Botswana Research Ethics Committee, the Office of Research and Development (Gaborone, Botswana), and the Health Research and Development Division of the Botswana MOH. Permissions were also obtained from heads of health facilities before recruitment of providers and women living with HIV took place. Since the only record linking the participant and the research would be the consent document, we received a waiver of documentation of written informed consent in order to fully protect the identities of all study participants. However, all participants provided comprehensive verbal informed consent.

## Author Contributions

SG, GH, and CM: conceived and designed the study. SG, NM, and CB: performed the experiments. SG, GH, NM, CB, and CM: analyzed the data. SG, GH, JH, DR-M, and CM: wrote the paper. All authors contributed to the article and approved the submitted version.

## Conflict of Interest

The authors declare that the research was conducted in the absence of any commercial or financial relationships that could be construed as a potential conflict of interest.
